# Nutritional assessment in dialysis patients: Much more than body composition

**DOI:** 10.1590/2175-8239-JBN-2021-0279

**Published:** 2022-02-21

**Authors:** Carla Maria Avesani, Alice Sabatino

**Affiliations:** 1 Karolinska Institute, Department of Clinical Science, Technology and Intervention, Division of Renal Medicine and Baxter Novum, Stockholm, Sweden.; 2 Karolinska University Hospital, Medical Unit Clinical Nutrition, Stockholm, Sweden.; 3 Parma University Hospital, Division of Nephrology, Parma, Italy.

The assessment of nutritional status in patients with chronic kidney disease (CKD) is one of the pillars to provide adequate nutritional care. An adequate nutritional assessment will guide the clinical practitioner, especially the dietitian, to plan the dietary goals, the strategies for intervention, the follow-up, and a new nutritional assessment. Choosing the method and time interval that provides the best balance of accuracy, reliability, and ease of use of tools for routine nutritional care remains a challenge and topic of debate. For this reason, a significant body of studies has been published in the last decades aiming to find the most suitable method for clinical and research purposes to provide adequate and accurate diagnosis of nutritional status^1, 2^.

In this regard, the study published in the current issue of the Brazilian Journal of Nephrology brings valuable information. Sugizaki et al.^3^ showed that nutritional status assessed by bioelectrical impedance vector analysis (BIVA) can be used as a complementary method to evaluate nutritional status and, in particular, the hydration status in patients on chronic hemodialysis (HD). The study enrolled 104 patients on HD, and the assessment of nutritional status by BIVA had a low sensitivity (true positive) but a good specificity (true negative) to diagnose malnutrition using the 7-point subjective global assessment (7p-SGA) as a reference method. The reason for this finding can be explained by differences between the two methods. BIVA aims to evaluate cellularity and hydration status based on body cell mass (BCM), which in turn includes all metabolically active tissues in the body, comprising muscle, internal organs, intra- and extracellular water^4^. The 7p-SGA, on the other hand, evaluates nutritional status by considering aspects related to spontaneous loss of body weight and appetite, changes in functional status and gastrointestinal tract, presence of comorbidities, and loss of muscle mass and subcutaneous fat on physical exam^5^. In other words, since both methods evaluate different aspects of nutritional status, a complete agreement between them would not be expected.

However, an important finding of the study by Sugizaki et al^3^ should not be overlooked. They showed a shift in the position of individual vectors of BIVA before and after the dialysis session due to an increase in Z (resistance) and reactance, indicating the removal of extracellular fluid. Of note, after the dialysis session, a shift to the low hydration quadrant was observed in all patients (both well-nourished and malnourished). Dehydration is a condition that is often overlooked in this clinical setting. In this regard, vector analysis by BIVA before and after dialysis can be used in association with other clinical aspects, including intradialytic and interdialytic body weight change, blood pressure change, and overall patient well-being, to better determine the ideal dry body weight of patients on HD. In addition, when comparing BIVA results between well-nourished and malnourished patients classified according to the 7p-SGA, a different value for cellularity markers was found, indicating the important role of BIVA as a complementary assessment of nutritional status, mainly for protein mass.

Much has been learned about assessment of nutritional status of patients on HD. One of the most important lessons is that there is no single method that provides a complete overview of nutritional status, as is evident in the Kidney Disease Outcome Quality Initiative (KDOQI) clinical practice guideline for nutrition in CKD published in 2020^2^. Rather, a combination of methods should be adopted. The choice of methods depends on the purpose: for routine assessment or for research. The publication by Sugizaki et al.^3^ provides evidence-based practice that supports the use of 7p-SGA and BIVA to assess nutritional and hydration status taking into account the clinical condition of each patient.
